# Drug utilization study of antiparkinsonian medication in Romania during 25 years

**DOI:** 10.3389/fphar.2025.1534344

**Published:** 2025-02-05

**Authors:** Camelia Bucsa, Denisa Bruhs, Anamaria Apan, Elena Francu, Cristina Mogosan, Irina Iaru

**Affiliations:** ^1^ Pharmacovigilance Research Center, Faculty of Pharmacy, “Iuliu Hatieganu” University of Medicine and Pharmacy, Cluj-Napoca, Romania; ^2^ Department of Pharmacology, Physiology and Pathophysiology, Faculty of Pharmacy, “Iuliu Hatieganu” University of Medicine and Pharmacy, Cluj-Napoca, Romania; ^3^ Neurology Medical Office Dr. Elena Ovidia Francu, Turda, Romania

**Keywords:** drug utilization study, antiparkinsonian medication, Romania, anti-Parkinson drugs, Parkinson’s disease, levodopa, dopamine agonist, anticholinergics

## Abstract

**Background:**

Antiparkinsonian medication has significantly evolved over the last 2 decades, offering various pharmacologic approaches. The aim of this study was to explore the trends and to determine the statistical significance of the observed changes in the antiparkinsonian medication utilization in Romania during 1998–2022.

**Methods:**

This antiparkinsonian drug utilization study used data provided by CEGEDIM Romania, originating from the Pharma and Hospital Report. Quantitative data for each ATC N04 antiparkinsonian medication were converted to total defined daily doses (DDDs) and to DDD/1000inhabitants/day (DDD/TID). The autoregressive integrated moving average (ARIMA) model was employed to determine the statistical significance of the observed changes in the trends of antiparkinsonian drug use.

**Results:**

The utilization of antiparkinsonian medication increased considerably (6-folds) in Romania during the 25 years, from 1.03 DDD/TID in 1998 to 6.22 DDD/TID in 2022. Starting 2005, dopamine precursor (levodopa) became the most used antiparkinsonian drug and remained on this position until the end of the study (13-fold increase from 0.17 in 1998 to 2.30 DDD/TID in 2022). MAO-B inhibitors represented the second most used antiparkinsonian drug class for the majority of the years. Selegiline was the most used until 2017 (0.82 DDD/TID), when a decrease in use was observed and continued until 2022 (0.49 DDD/TID). Utilization of dopamine agonists started in 1999, with less than 0.01 DDD/TID, and increased to 1.47 DDD/TID in 2022. Ropinirole was the most used dopamine agonist (0.56 DDD/TID in 2022). Anticholinergic agents represented the most used antiparkinsonian drugs until 2005. Trihexyphenidyl was the main anticholinergic prescribed with a maximum utilization of 0.82 DDD/TID in 2000 followed by a slight decrease until 2022 (0.56 DDD/TID). Amantadine utilization was mainly constant throughout the 25 years, with 0.32 DDD/TID prescribed in 2022. ARIMA analysis showed that the changes in antiparkinsonian drugs consumption were not statistically significant and overall, the trend for antiparkinsonian drug use demonstrates an upward trajectory.

**Conclusion:**

Antiparkinsonian medication showed an increasing utilization trend in Romania during 1998–2022. Levodopa was the most used antiparkinsonian medicine after 2005, replacing anticholinergic agents. MAO-inhibitors utilization came in second and was followed by dopamine agonists. Observing the trend in antiparkinsonian medication utilization over time is essential for providing insights into their real-world use and uptake in a large population.

## 1 Introduction

The prevalence of Parkinson’s disease (PD) has significantly increased in the last decade worldwide, being the second most common neurodegenerative disease (Feigin et al., 2017; Olesen et al., 2012). In Europe, 1.2 million patients were diagnosed with PD in 2010, reaching a prevalence of 1,838,098 in 2017 (Olesen et al., 2012; Deuschl et al., 2020). In Romania, recent data showed that more than 70,000 patients (approximately 379 people per 100,000) are affected by this disease (Szasz et al., 2020; Rosca et al., 2021). This data is likely underestimated due to underdiagnosis, which remains a challenge even today. There is no specific test for the diagnosis of PD, especially in the early stages, and the diagnosis is mostly based on clinical criteria. Moreover, the clinical features of PD are varied and often overlap with other neurodegenerative conditions (Tolosa et al., 2021; Jankovic, 2008).

PD is characterized by the degeneration of dopamine-producing neurons in the substantia nigra and manifests with slowness of voluntary movements, tremors and non-motor symptoms (Simon et al., 2020). Pharmacologic agents are available for managing the symptoms of PD. Levodopa, dopamine agonists, monoamine oxidase B (MAOB) inhibitors, catechol-O-methyltransferase (COMT) inhibitors, anti-cholinergic agents, and amantadine are therapeutic options used to improve clinical outcomes (Church, 2021). Individualized treatment is employed for each patient diagnosed with PD taking into consideration the particularities of the patient, such as age, disease progression, symptoms, co-morbidities, co-medication, or previous adverse reactions to other drugs. However, there is a proportion of approximately 20% of PD diagnosed patients that do not receive medication (Dahodwala et al., 2016).

Observing the trend in antiparkinsonian medication utilization over time is essential for providing insights into the real-world use and uptake of these drugs in a large population. While different drug utilization studies (DUS) are available for various countries in Europe and worldwide (Kalilani et al., 2019; Brandt-Christensen et al., 2006; Leoni et al., 2002; Trifirò et al., 2008; Mitkova et al., 2021), there is limited data published about the utilization of antiparkinsonian medication at the national level and no information on the evolving trends over time, in Romania. A scoping review dedicated to the PD literature published for Romania showed a positive trend in PD research based on an increasing number of published studies. However, these studies tend to focus more on clinical data, and less on the interventions, pharmacological or non-pharmacological (Rosca et al., 2021). Regarding the PD pharmacological approaches, a cross-sectional study that included 1,237 hospitalized PD patients in a county hospital in Romania, looked at the therapeutic options used in the early stages of the disease (disease duration <5 years). The main findings were that 81% of the patients were treated with levodopa alone or in combination (Szász et al., 2019). Another study including 95 Romanian patients with advanced PD showed a percentage of 84% of patients on levodopa, followed by 56% on MAO-B inhibitors, and 38% on dopamine agonists (Szasz et al., 2021).

The prescribing patterns in PD may be affected by several factors, including changes in treatment guidelines. In 2009 Parkinson’s guidelines from Romania were recommending treatment initiation with dopamine agonists and only after the effectiveness of these agents wares off, levodopa should be introduced into therapy (MS, 2025). These recommendations were based on the results of the studies from the early 2000s that showed potential neuroprotective effects of dopamine agonists (Orayj and Lane, 2019). However, these properties were not confirmed and in 2006 the American Association of Neurology (Suchowersky et al., 2006a), and in 2010 Romanian guidelines (Neurology, 2025b), recommend starting therapy with dopamine agonists or other dopaminergic therapies (MAO-B inhibitors or levodopa) in the early stages of PD if the motor symptoms do not impact patients’ quality of life (QoL). When QoL was investigated in the 2014 PD-MED study, it was found that early initiation of levodopa resulted in a better QoL on the long term than initiating dopamine agonists and MAO-B inhibitors (PD MED Collaborative Group, 2014).

Safety concerns could significantly influence the prescribing patterns in PD patients. The ergot-derived dopamine agonists are well-known for the risk of development of the symptoms of fibrosis and fibrotic changes in cardiac valves, risk that was not demonstrated for non–ergot-derived dopamine agonists. Moreover, the use of ergot-derived dopamine agonists was further restricted after 2008 when they were associated with an increased risk of fibrosis in patients under chronic treatment, suggesting that fibrosis can start to develop far before the occurrence of symptoms (Zanettini et al., 2007; Ema, 2025). After 2011, the use of dopamine agonists was affected by the concerns of impulse control disorders related to their use (Orayj and Lane, 2019). The risk of serotonin syndrome under rasagiline is minimal compared to selegiline. At lower doses, the selectivity of selegiline for MAO type A decreases the risk of drug-drug interactions or drug-food interactions, however, when using higher doses, the selectivity is lost, both subtypes of MAO, A and B being inhibited, with increased risk of hypertensive crises when selegiline is used in association with amine-containing foods or certain drugs (such as serotonin reuptake inhibitors) (Csoti et al., 2012). In case of tolcapone, a first case report was published in 1998, describing the fatal case of acute liver failure attributed to tolcapone utilization (Assal et al., 1998). In 2020, three more cases of acute liver failure after tolcapone utilization were reported, two of these cases being fatal (Olanow, 2000). Therefore, liver function tests are now strictly recommended, and tolcapone is contraindicated for patients with liver disease (Author Anonymous, 2025e). In this context of PD treatment challenges the present study aims to explore the trends and to determine the statistical significance of the observed changes in antiparkinsonian medication utilization in Romania during 1998–2022.

## 2 Materials and methods

### 2.1 General considerations for antiparkinsonian medication prescribing in Romania

The antiparkinsonian medication is mainly prescribed through the national social health insurance (SHI) system in Romania, which covers nearly 90% of the population. The cost is reimbursed by the National Health Insurance House (NHIH), if prescribers follow national regulations and protocols published by the Ministry of Health and NHIH and use specific approved diagnostic codes. Medicines can be found on the List of reimbursement medications (LRM) with different reimbursement percentages from the reference price, depending on criteria such as medicine status (innovative or generic), type of the targeted disease, or available national health programs. Up to three different types of antiparkinsonian medications per month are fully reimbursed by the NHIH regardless of the medicine status (Author Anonymous, 2025b).

In outpatient care, only neurologists can initiate and adjust the PD treatment and family medicine (FM) physicians can continue the prescribing. FM physicians have a gatekeeping role in primary care, although patients can directly access specialists. In inpatient care, neurologists are the specialists who can prescribe antiparkinsonian medication for the treatment of Parkinson disease. Neurologists, psychiatrists, gastroenterologists, and physical therapists form a multidisciplinary team that oversees the treatment plan of PD patients (Author Anonymous, 2025b).

Regarding the national LRM and the levels of coverage for antiparkinsonian medication in 2022, most antiparkinsonian medication had a 100% reimbursement level. The prescription of antiparkinsonian medication in Romania was carried out based on the therapeutic protocols developed by the specialized commissions of the Ministry of Health. For apomorphine, the treatment could be carried out through the approved cost-volume contracts (Author Anonymous, 2025a; Author Anonymous, 2025c).

In this study, antiparkinsonian medication total utilization is depicted, meaning that the use of the specific medication in other diseases is comprised. These medications are amantadine also prescribed for antiviral treatment, trihexyphenidyl also prescribed in case of mental illnesses for the control of extrapyramidal symptoms, and other off-label use such as selegiline in depression.

Pramipexole and piribedil had a 50% reimbursement level in the LRM. Pramipexole treatment could be initiated by the specialist and further prescribed by the FM based on the medical letter issued by the specialist. Piribedil treatment could be carried out based on the national therapeutic protocols developed by the specialized commissions of the Ministry of Health (Author Anonymous, 2025a; Author Anonymous, 2025c).

### 2.2 Data source

A retrospective descriptive study was conducted to investigate the use of dispensed antiparkinsonian drugs in Romania from January 1998 to December 2022.

The data used in this study was provided by the Management Center for Documentation, Information and Marketing (CEGEDIM) Romania. CEGEDIM Romania is a company that provides specialized software, databases, and data flow management for the healthcare industry. Given the fact that the National Agency for Medicines and Medical Devices of Romania (NAMMDR) does not hold a national database with drug consumption, CEGEDIM Romania is the provider of such data for NAMMDR, facilitating activities such as monitoring the prescription of medicines (Author Anonymous, 2025d).

Consumption data was obtained from the CEGEDIM Pharma and Hospital Report study, and covered a number of 4,700 retail pharmacies and 75 hospital pharmacies (representing over 60% of retail and 18% of hospital pharmacies in Romania). At a 95% confidence level, the error margin for national data extrapolation was ±1% for retail and ±10% for hospital pharmacies.

Antiparkinsonian drugs from the Anatomical Therapeutic Chemical (ATC) code level 4 (ATC N04) class were included in the analysis (Table 1). Opicapone and safinamide use was not reported in Romania during the study period and were not included. The following information was available for each antiparkinsonian agent: administration route, strength (mg/dose), number of doses per package, number of packages (units) dispensed each year and the manufacturer.

**TABLE 1:** Antiparkinsonian drugs included in this study.

Pharmacologic class	ATC code	Antiparkinsonian agent	DDD^a^
Dopamine precursor	N04BA02	Levodopa + carbidopa	0.6 g
	N04BA02	Levodopa + benserazide	0.6 g
	N04BA03	Levodopa + carbidopa + entacapone	0.45 g
Dopamine agonists	N04BC04	Ropinirole	6 mg
	N04BC05	Pramipexole	2.5 mg
	N04BC07	Apomorphine	20 mg
	N04BC08	Piribedil	0.2 g
	N04BC09	Rotigotine	6 mg
COMT inhibitors	N04BX01	Tolcapone	0.45 g
	N04BX02	Entacapone	1 g
MAO-B inhibitors	N04BD01	Selegiline	5 mg
	N04BD02	Rasagiline	1 mg
Adamantan derivatives	N04BB01	Amantadine	0.2 g
Anticholinergic agents	N04AA01	Trihexyphenidyl	10 mg
	N04AA02	Biperiden	10 mg

^a^ According to World Health Organization (WHO) (WHO, 2025).

### 2.3 Data analysis

Defined daily doses (DDDs) method was chosen to quantify antiparkinsonian consumption in Romania. The reason behind this choice relies on the type of data provided by CEGEDIM which allowed for quantitative data of each antiparkinsonian agent to be converted into total DDDs and number of DDDs/1000inhabitants/day (DDD/TID). According to the World Health Organization Collaborating Centre for Drug Statistics Methodology, DDD is defined as “the assumed average maintenance dose per day for a drug used for its main indication in adults”. The DDDs for each drug used in this study were extracted from the 2023 edition of the WHO ATC/DDD Index and are included in Table 1 (WHO, 2025). Moreover, DDDs method enables longitudinal studies to identify trends in drug use over time, and provides a consistent unit of measurement that enables comparisons of drug use across different regions, populations, and time periods. Individual patient-level data or prescription data were not available for the purpose of this study.

Total DDDs were calculated for each medication by applying the following formula:

$$total\;DDDs=\frac{number\;of\;packages\times number\;of\;doses\;per\;package\times number\;ofmg\;per\;dose}{WHO\;DDD\;\left(mg\right)}$$

In order to calculate the DDD/TID, two more variables were considered: the estimated population in Romania each year and the number of days per year. The number of inhabitants per year was obtained from the National Institute of Statistics for the period 1998–2013 and from the Eurostat database, the statistical office of the European Union for the period 2013–2022 (National Institute of Statistics, 2020; European Commission, 2023).

DDD/TID was calculated, for each medication per year, by applying the following formula:

$$DDD/TID=\frac{total\;DDDs\times 1000}{number\;of\;inhabitants\;in\;Romania\;for\;the\;year\times number\;of\;days\;in\;the\;year}$$

For this study, the use of dopamine precursor levodopa was calculated altogether, without distinction between the different combinations (levodopa + carbidopa, levodopa + benserazide, and levodopa + carbidopa + entacapone).

### 2.4 Statistical analysis

We used the ARIMA (Auto Regressive Integrated Moving Average) model to predict the prescribing trends to determine the statistical significance of the observed changes. The ARIMA model, was selected for its versatility in handling various components of a time series.

A prerequisite for ARIMA modeling is data stationarity. Autocorrelation function (ACF) and partial autocorrelation function (PACF) tests were applied to total antiparkinsonian drugs utilization. First-order differencing made the time series stationary, with the goal of removing trends from the data, so that the time series becomes more predictable. Therefore, the ARIMA (0,1,0) model, commonly known as a random walk model, emerged as the most appropriate model for capturing trends across all antiparkinsonian drugs in the present study (Schaffer et al., 2021). Analyses were performed using the SPSS Version 23 statistic software package. All descriptive analysis was performed using Microsoft Office Excel 2012.

## 3 Results

### 3.1 Total utilization of antiparkinsonian medication

**FIGURE 1:**
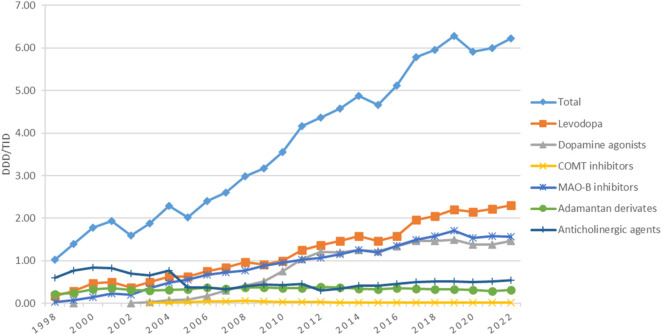
Use of antiparkinsonian medication in Romania during 1998–2022, total and by pharmacologic class.

The utilization of antiparkinsonian medication increased considerably during the study period, from 1.03 DDD/TID in 1998 to 6.22 DDD/TID in 2022, an overall increase of 6-folds (Figure 1).

### 3.2 Utilization of levodopa

Utilization of levodopa increased more than 13-fold, from 0.17 in 1998 to 2.30 DDD/TID in 2022. Starting 2005, levodopa became the most used antiparkinsonian drug and remained in this position until the end of the study. During the 1998–2022 period, small decreases in the utilization of levodopa were noticed in 2002, 2009, and 2015, but the usage continued to grow in the years after (Figure 1).

### 3.3 Utilization of dopamine agonists

**FIGURE 2:**
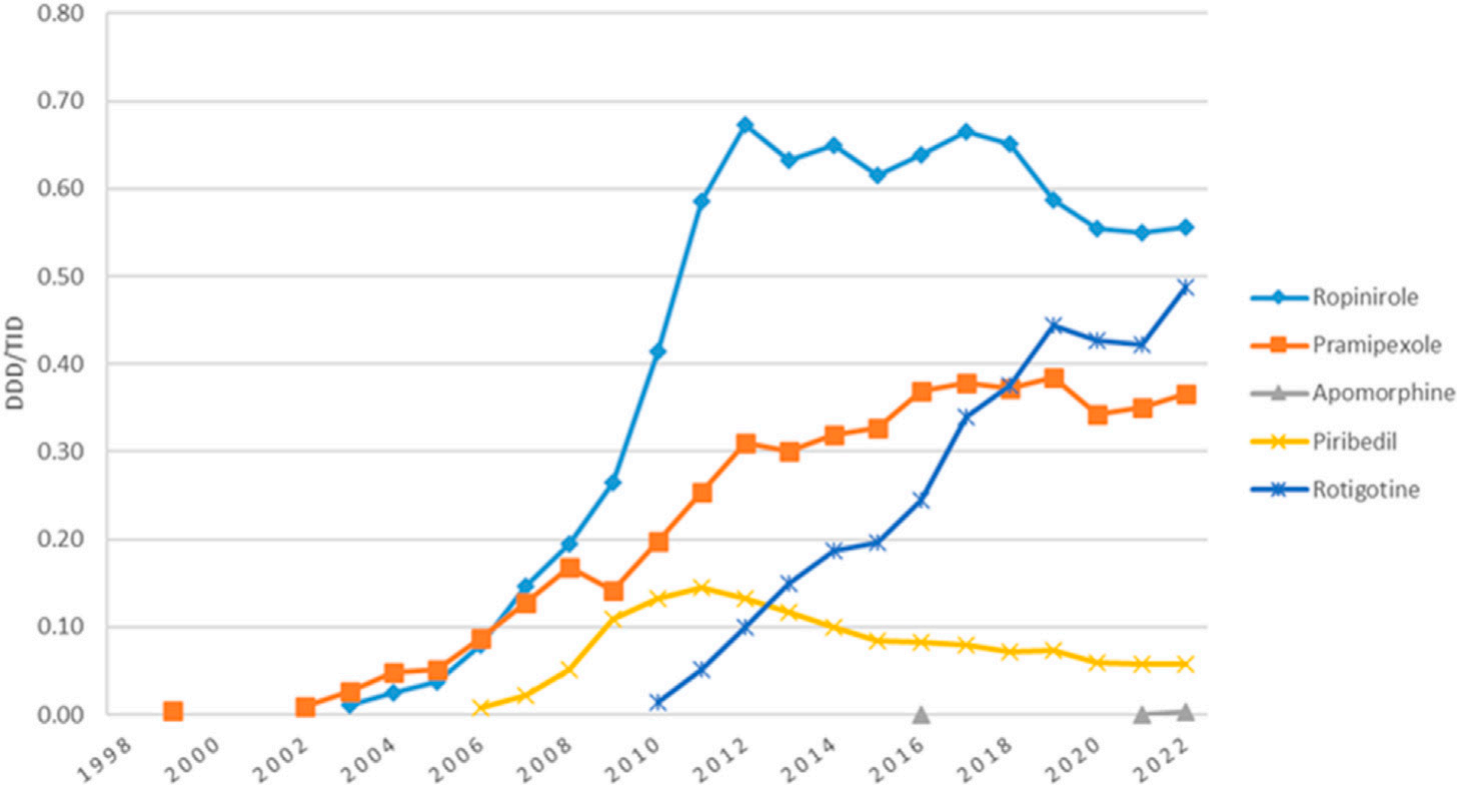
Use of individual dopamine agonists drugs during 1998–2022.

Utilization of dopamine agonists was first reported in 1999, with less than 0.01 DDD/TID, and increased to 1.47 DDD/TID in 2022, when they represented the second most used antiparkinsonian class. However, in most years, they were the third most used, after levodopa and MAO-B inhibitors (Figure 1). Figure 2 shows the utilization of each individual dopamine agonist during the study period. Ropinirole was the most used dopamine agonist. Its use increased at a fast pace from 2003 (0.01 DDD/TID) to 2012 (0.67 DDD/TID), and then slightly decreased until 2022 (0.56 DDD/TID). Pramipexole was the second most used dopamine agonist until 2018 when rotigotine took its place until the end of the study period. A lower utilization was observed for piribedil, with an increase until 2012 and a slight decrease afterward. However, the lowest utilization was observed for apomorphine, for which the use was reported during 3 years (2016, 2021, and 2022) of the 25 years duration of the study, with less than 0.01 DDD/TID each year.

### 3.4 Utilization of COMT inhibitors

COMT inhibitors represented the lowest-used class of antiparkinsonian drugs, with a slight increase from 2003 (0.01 DDD/TID) to 2008 (0.06 DDD/TID), followed by a decrease until 2022 (0.02 DDD/TID) (Figure 1). This is mainly owed to the utilization of entacapone alone, since tolcapone was only used for the first 2 years of the study (<0.01 DDD/TID for each of the 2 years).

### 3.5 Utilization of MAO-B inhibitors

**FIGURE 3:**
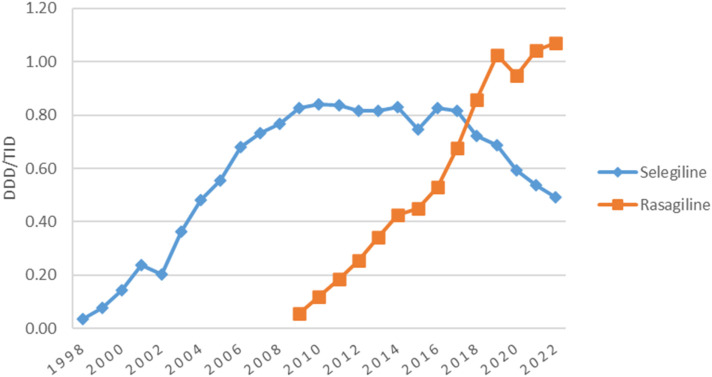
Use of individual MAO-B inhibitors drugs during 1998–2022.

MAO-B inhibitors represented the second most used antiparkinsonian drugs class for the majority of the years (Figure 1). Their utilization increased continuously from 1998 (0.04 DDD/TID) to 2019 (1.71 DDD/TID), decreased thereafter until 2020 (1.54 DDD/TID), and then remained stable until 2022. Figure 3 shows the trend in MAO-B inhibitors utilization during the study period. Selegiline was the most used MAO-B inhibitor until 2017 (0.82 DDD/TID) and then decreased until 2022 (0.49 DDD/TID). Rasagiline utilization was first reported in 2009 and markedly increased until the end of the study (1.07 DDD/TID).

### 3.6 Utilization of adamantan derivatives

The utilization of amantadine was mainly stable during the entire study period (Figure 1). Its utilization increased from 0.02 DDD/TID in 1998 and reached a peak of 0.37 DDD/TID in 2006. Thereafter, amantadine utilization was mainly constant, with a very small decrease to 0.32 DDD/TID in 2022.

### 3.7 Utilization of anticholinergic agents

**FIGURE 4:**
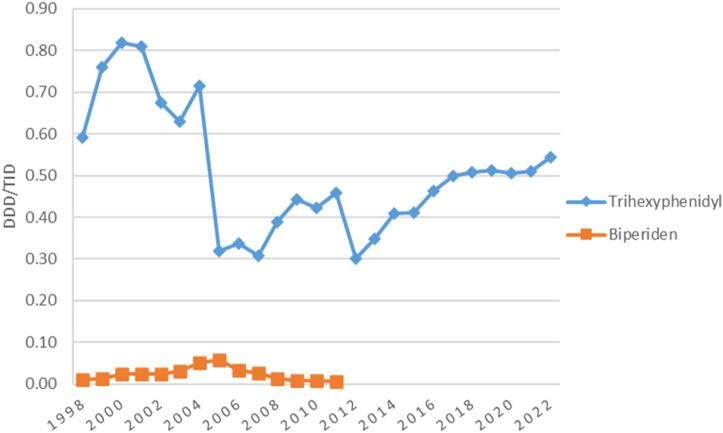
Use of individual anticholinergic agents during 1998–2022.

The utilization of anticholinergic agents was mainly the same in the first year (0.60 DDD/TID) and the last year (0.56 DDD/TID) of the study, with small fluctuations in the years in between. They represented the most used antiparkinsonian drugs until 2005 when levodopa took the lead (Figure 1). Figure 4 shows that the most used anticholinergic agent was by far trihexyphenidyl. The utilization of trihexyphenidyl increased from 0.59 DDD/TID in 1998 to a maximum utilization of 0.82 DDD/TID in 2000. Thereafter, a marked drop was observed in 2005 (0.32 DDD/TID) and in 2012 (0.30 DDD/TID), followed by an increase until 2022 (0.56 DDD/TID). The utilization of biperiden slowly increased from 1998 (0.01 DDD/TID) until 2005 (0.06 DDD/TID), then decreased until 2012, when its utilization stopped being reported.

### 3.8 ARIMA analysis

Overall, an increasing trend in drug consumption over the years has been observed. ACF and PACF confirmed the non-stationarity of the data (Supplementary Figures S1, S2). Supplementary Figure S3 shows the series after single differentiating when the increasing trend was eliminated.

**FIGURE 5:**
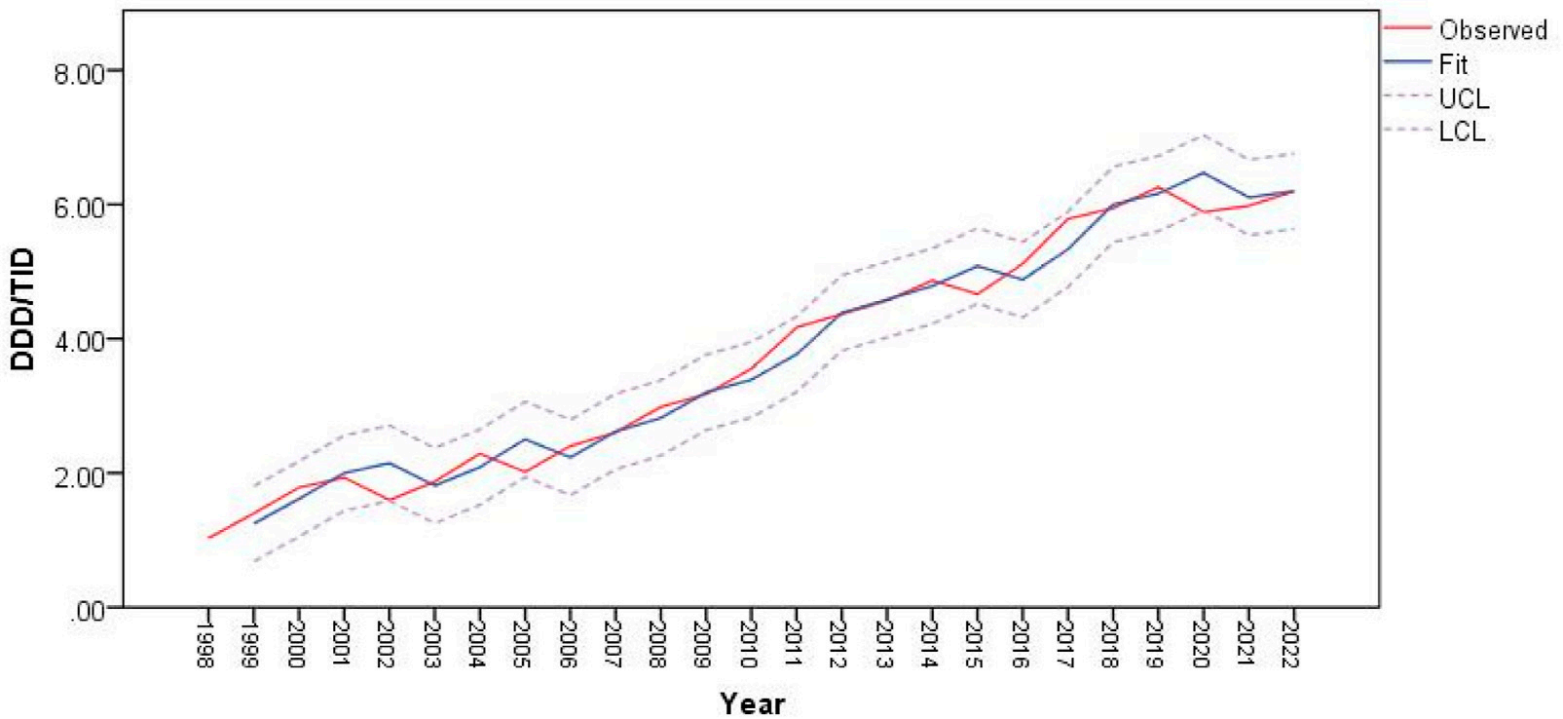
Predictions for antiparkinsonian drug utilization (expressed as DDD/TID) using ARIMA (0,1,0) model.

Figure 5 shows that the ARIMA (0,1,0) model fits the data well in terms of capturing antiparkinsonian drugs utilization patterns, with the residuals appearing to be white noise.

In 2002, 2005, 2015 and 2020, the predicted values of drug utilization are increased, compared to the actual observations. However, these increases fit within the upper and lower confidence interval limits of the ARIMA model, along with the observed values of antiparkinsonian drugs use. The decrease in antiparkinsonian drugs consumption in those years was not statistically significant and overall, the trend for antiparkinsonian drug use demonstrates an upward trajectory.

## 4 Discussion

### 4.1 Total utilization of antiparkinsonian medication

The antiparkinsonian medication utilization in Romania was in continuous growth from 1998 to 2022, with a 6-fold increase between the first and the last year of the period (from 1.03 DDD/TID in 1998 to 6.22 DDD/TID in 2022). This is in line with the increase in the number of PD patients in the last 25 years worldwide, in Romania reaching 72,000 in 2021 (379 people per 100,000) (Rosca et al., 2021). The PD prevalence in Romania is mostly based on the number of PD patients who are on prescribed medication (Orayj and Lane, 2019). The increase in prevalence (40,517 PD patients in 2016) (Neurology, 2025a) could be explained by improved diagnosis rates (due to improvements in the general population health education, increased accessibility to health services and treatment, continuous medical education, and family physicians awareness of PD), an increase in the proportion of patients receiving treatment and increased life expectancy. Changes in guidelines and increased accessibility to treatment are other determinants that may contribute to the increase in the prescribing of medication (Orayj and Lane, 2019).

Along the 25-year ascending line we observed decreases in the utilization of the antiparkinsonian medication in 2002, 2005, 2015, and 2020 (Figure 1). In addition to the changes in the PD guidelines (already described in the introduction section), major changes happened in Romanian health system over the study period that could have impacted the prescribing in chronic diseases. In 2002, a major healthcare system reorganization occurred when for the first time each citizen was free to address any medical facility (i.e., physician office, hospital) of his/her choice; also, the first predefined package of medical services was established (Spiru et al., 2011). Further, in 2005, a significant change regarded primary health assistance, the family physician became the first point of contact for the citizens within the public health system. It became also mandatory to be enrolled with a family physician’s office to access health services (Purcărea et al., 2015). This change may have led to delays in the prescribing of the antiparkinsonian medication due to time needed for FM enrollment. In 2015 a 20%–30% price reduction was applied to prescription medicines starting 1^st^ of July 2015 (Legislatie, 2024a). This regulation led to Romania having the lowest prices for medications in Europe, which in turn led to medication export. This touched the entire production-distribution-retail chain in the pharmaceutical industry leading to supply issues for the Romanian market. The shortage of levodopa/benserazide from the Romanian market due to parallel export was mentioned in the national press at the beginning of the 2016 (Digi24, 2025). The beginning of 2020 came with the COVID-19 pandemics, a major challenge for the healthcare system. A decrease in the utilization of medicines was noticed in Romania in 2020 (Bircea and Teodora, 2022). An explanation could be the emergency and alert state that limited access to health care for chronic diseases. We hypothesize that, as for all chronic diseases, many health services were missed or delayed in Parkinson patients, and new prescriptions for newly diagnosed patients with Parkinson decreased. The ARIMA (0,1,0) model suggested that drug utilization trends for antiparkinsonian drugs showed a general upward trajectory over time, although there are the years 2002, 2005, 2015, and 2020 where the predicted values were higher than actual observations. The potential factors discussed above for the specific years might have affected the actual consumption. However, these discrepancies are within the confidence intervals of the model, meaning that the model’s prediction for those years were reasonable, even if the exact values did not match the observed values. Moreover, the decreases in drug consumption in those specific years were not statistically significant, indicating that these drops were short-term variations that do not indicate a long-term change in the pattern of drug usage.

In the first years of the study, the total antiparkinsonian medication utilization in Romania was higher than the utilization in Croatia (about 0.78 DDD/TID versus 1.79 DDD/TID in Romania in 2000) (Brkicic et al., 2012), and lower compared to countries such as New Zealand (1.48 DDD/TID versus 1.03 DDD/TID in Romania in 1998) (Pitcher et al., 2014), Spain (3.85 DDD/TID in 1998) (Osinaga et al., 2007) and Australia (2.81 DDD/TID versus 1.60 DDD/TID in Romania in 2002) (Hollingworth et al., 2011). In the last years of the study, antiparkinsonian medication consumption in Romania was higher than the utilization in other countries such as Norway (4.19 DDD/TID) (Berg et al., 2022) and Finland (5.21 DDD/TID) (Finnish Medicines Agency Fimea, 2021), versus 6.00 DDD/TID in Romania in 2021, and Estonia (3.70 DDD/TID versus 6.22 DDD/TID în Romania în 2022) (Statistics on Medicines, 2024).

### 4.2 Utilization of levodopa

For the majority of the years, levodopa was the most used antiparkinsonian agent in Romania. Decreases in the utilization of levodopa coincided with the ones of the overall antiparkinsonian medication except for the 2009 decrease. This could be explained by the inclusion on the 2009 LRM of several generics for ropinirole, with a sharp increase in ropinirole utilization that may have replaced a part of levodopa market share. A decrease in levodopa utilization was also observed in Japan and Croatia in 2009. The authors also attributed this change to the introduction of several new dopamine agonist agents and their inclusion on the health insurance reimbursement list (Nakaoka et al., 2014; Suzuki et al., 2020; Brkicic et al., 2012).

Other events that may have affected levodopa’s utilization are the recommendations of the national guidelines early in 2009 to use dopamine agonists (mostly pramipexole, ropinirole, rotigotine) in the early stage of the disease (MS, 2025) due to their neuroprotective effect (studies further refuted this effect in 2011–2013) (Orayj and Lane, 2019). Additionally, in the early 2000s, multiple studies reported that long-term levodopa could contribute to neurotoxicity (Mytilineou et al., 2003; Jankovic, 2000). These findings were further refuted and the American Academy of Neurology (AAN) guidelines in 2006 stated that levodopa does not accelerate disease progression (Suchowersky et al., 2006b).

The same leading place of levodopa was observed in other European countries (Orayj and Lane, 2019), such as Spain (Osinaga et al., 2007), Croatia (Brkicic et al., 2012), Italy (Trifirò et al., 2008), United Kingdom (United Kingdom) (Kalilani et al., 2019), and Bulgaria (Mitkova et al., 2021), but also for Asian countries (Orayj and Lane, 2019) such as Taiwan (Liu et al., 2016), Japan (Nakaoka et al., 2014; Suzuki et al., 2020), and China (Yi et al., 2022), for South Africa (Gaida and Truter, 2014), and United States of America (United States of America) (Kalilani et al., 2019; Crispo et al., 2015). In the first years of the study, in Romania, levodopa utilization (0.18 DDD/TID in 1998) was lower than the utilization in New Zealand (0.84 DDD/TID) (Pitcher et al., 2014) and Spain (1.68 DDD/TID) (Osinaga et al., 2007). In the year 2000, we also found a lower utilization (0.47 DDD/TID) than Croatia (0.59 DDD/TID) (Brkicic et al., 2012). However, in the last years of the study, Romania had a higher consumption of levodopa than Latvia (1.02 DDD/TID versus 2.04 DDD/TID in Romania in 2018) (Statistics on Medicines Consumption, 2024), Bulgaria (1.33 DDD/TID versus 2.21 DDD/TID Romania in 2019) (Mitkova et al., 2021), Norway (1.89 DDD/TID versus 2.22 DDD/TID in Romania in 2021) (Berg et al., 2022) Estonia (1.49 DDD/TID versus 2.31 DDD/TID in Romania in 2022) (Statistics on Medicines, 2024).

### 4.3 Utilization of dopamine agonists

Dopamine agonists utilization in Romania increased from 0.01 DDD/TID in 1999 to 1.47 DDD/TID in 2022 and similarly to other countries (Orayj and Lane, 2019), such as Italy (Trifirò et al., 2008), Australia (Hollingworth et al., 2011), Spain (Osinaga et al., 2007), and Japan (Nakaoka et al., 2014; Suzuki et al., 2020), they represented the second or third most used antiparkinsonian medication class (alternatively with MAO-B) for most of the time during 1998–2022. However, while in most countries dopamine agonists utilization decreased after 2011 (Orayj and Lane, 2019) due to raising concerns about the impulse control disorders associated with this class (Orayj and Lane, 2019; Hollingworth et al., 2011), this was not the case in our data.

By far, the most used dopamine agonist in Romania was ropinirole, almost for the entire period of the study, and the same results were found in Spain (Osinaga et al., 2007), New Zealand (Pitcher et al., 2014), Croatia (Brkicic et al., 2012), Finland (Finnish Medicines Agency Fimea, 2021), Latvia (Statistics on Medicines Consumption, 2024), and United States of America (Kalilani et al., 2019). However, other countries such as United Kingdom (Dahodwala et al., 2016), Japan (Nakaoka et al., 2014; Suzuki et al., 2020), and Bulgaria (Mitkova et al., 2021) found the most used dopaminergic agonist to be pramipexole, which was the second most used in Romania. The 2009 drop in pramipexole may be due to concerns of impulse control disorders (gambling) but also due to parallel export that is suspected for levodopa too in the same year (Orayj and Lane, 2019; Digi24, 2025). Ropinirole utilization had a general descending trend with small fluctuations during 2011–2018 and slight decrease after 2019 that could be explained by the sharp increase in rotigotine prescription.

Starting August 2016, the use of piribedil was restricted for the treatment of Parkinson disease, as monotherapy (especially for tremorgenic forms) or associated with levodopa (from the start of the therapy or subsequently). This was the consequence of the benefit/risk balance assessment in France in 2013, showing the lack of evidence for piribedil to be indicated for other conditions (e.g., cognitive disorder, intermittent claudication) (Author Anonymous, 2016).

The low utilization of apomorphine in Romania can also be seen in other countries such as Spain (Osinaga et al., 2007), New Zealand (Pitcher et al., 2014), Australia (Hollingworth et al., 2011), Bulgaria (Mitkova et al., 2021) and Finland (Finnish Medicines Agency Fimea, 2021), where its utilization reached a maximum of 0.02 DDD/TID during the study intervals. In New Zealand, in contrast to Romania, apomorphine was used throughout each year of the study, with a notable increase in its utilization over time. Pitcher et al. attribute this increase to physicians becoming more familiar with the efficacy and technical aspects of apomorphine subcutaneous delivery, along with the provision of free infusion pumps by the marketing company (Pitcher et al., 2014). In 2020, apomorphine met the score of conditional inclusion in the LSM for the treatment of motor fluctuations (the “on-off” phenomenon) in patients with Parkinson’s disease insufficiently controlled by the administration of other antiparkinsonian drugs, and the cost-volume contract was concluded for the 2021–2022 period. Meanwhile, by the end of 2021, apomorphine was included in the LRM with non-conditioned status.

### 4.4 Utilization of COMT inhibitors

In Romania, COMT inhibitors utilization was very low during the 1998–2022 period. The utilization slowly increased in 2007 and 2008, due to the increase in entacapone consumption, but remained lower than any of the other antiparkinsonian classes. A similar utilization pattern for COMT inhibitors was noticed in Spain (Osinaga et al., 2007), Australia (Hollingworth et al., 2011), Croatia (Brkicic et al., 2012), Norway (Berg et al., 2022), and a very small difference in Finland (Finnish Medicines Agency Fimea, 2021), where entacapone utilization exceeded 0.03 DDD/TID. However, different results were found in New Zealand (Pitcher et al., 2014), where during 2007–2011, entacapone utilization was higher than in other countries, increasing from 0.05 DDD/TID to 0.09 DDD/TID. Pitcher et al. consider that this increase in entacapone utilization reflects the quick uptake of the agent once it was funded for use in New Zealand (Pitcher et al., 2014). While approved in 1997 in Europe (Orayj and Lane, 2019), tolcapone utilization in Romania was found in 1998 and 1999 only, possibly in relation to hepatotoxicity concerns.

### 4.5 Utilization of MAO-B inhibitors

MAO-B inhibitors utilization in Romania steadily increased during the study period and was higher compared to other countries, such as Bulgaria (0.31 DDD/TID versus 1.71 DDD/TID in Romania in 2019) (Mitkova et al., 2021), Norway and Finland (1.08 DDD/TID and 1.06 DDD/TID, respectively versus 1.58 DDD/TID in Romania in 2021) (Berg et al., 2022; Finnish Medicines Agency Fimea, 2021), and Latvia (0.36 DDD/TID versus 1.58 DDD/TID in Romania in 2018) (Statistics on Medicines Consumption, 2024). During this period, selegiline represented the second most used antiparkinsonian drug, after levodopa. Selegiline was also the second most prescribed antiparkinsonian drug in Spain (Osinaga et al., 2007).

Rasagiline utilization began in Romania in 2009 and continuously increased until the end of the study. Starting 2018, rasagiline became the most used MAO-B inhibitor in Romania as opposed to Australia (Hollingworth et al., 2011) and New Zealand (Pitcher et al., 2014) where it was not used, while in Estonia it was the only MAO-B inhibitor used (Statistics on Medicines, 2024). Rasagiline has gradually replaced selegiline in clinical practice, probably due to safety profile advantages and marketing strategies. However, it remains unclear whether selegiline metabolites can cause amphetamine-like adverse events, including cardiovascular and central neural system adverse events (Asano et al., 2023).

### 4.6 Utilization of anticholinergic agents

The evolution of anticholinergic utilization in Romania during the study period was mainly influenced by the safety issues, the uptake of other antiparkinsonian agents and by their use outside PD, such as the treatment of extrapyramidal side effects induced by antipsychotics.

The recommendation for anticholinergics utilization for PD in Romania is limited to younger patients presenting tremor as the main symptom, due to important side effects that could affect the older adult population. PD patients in general may be especially vulnerable to this medication, because it can commonly cause or exacerbate confusion and pose a risk for future dementia, and are potential candidates for deprescribing interventions (Nawaz et al., 2022). However, one study looking at prescription habits during 2018–2019 related to chronic pathologies of elderly people in primary care in Romania showed that trihexyphenidyl was used as a monotherapy for patients with Parkinson’s disease in 0.18% of cases (a misuse of medicines according to Beers 2019 Criteria) (Buda et al., 2021).

Until 2005, anticholinergic agents were the most used class of antiparkinsonian medication, with the maximum utilization of 0.84 DDD/TID in 2000. We found two drops in trihexyphenidyl use. One in 2005–2007 (0.32–0.30 DDD/TID) which may be explained partly by the increasing uptake of other pharmacologic classes and partly by the administrative changes in the primary health assistance (Szasz et al., 2020). The one in 2012 (0.30 DDD/TID) was caused by the market deficit.

A decrease in anticholinergics utilization was also noticed in other countries (Orayj and Lane, 2019), in New Zealand (from 1.40 DDD/TID in 1995 to 0.72 DDD/TID in 2011) (Pitcher et al., 2014) and Spain (from 1.02 DDD/TID in 1992 to 0.70 DDD/TID in 2004) (Osinaga et al., 2007). The authors believe that these drugs are not prescribed as often anymore due to the existence of other pharmacological alternatives with a better benefit-risk balance (Osinaga et al., 2007). However, the utilization in these countries was higher than the utilization in Romania. On the other side, the utilization in Norway (0.13 DDD/TID vs. 0.54 DDD/TID in Romania in 2021) and Finland (0.21 DDD/TID vs. 0.54 DDD/TID in Romania in 2021) was lower compared to Romania (Berg et al., 2022; Finnish Medicines Agency Fimea, 2021).

The most used anticholinergic agent in Romania during the study period was trihexyphenidyl, while biperiden utilization was very low until 2012 when its utilization in Romania stopped being reported. Different results were seen in Spain (Osinaga et al., 2007), Australia (Hollingworth et al., 2011) and Norway (Berg et al., 2022), where the most used anticholinergic agent was biperiden, its utilization being significantly higher than the utilization in Romania. Other countries, such as Bulgaria and Finland only used biperiden as an anticholinergic agent, while Estonia only used trihexyphenidyl, with the same utilization in 2022 as it was found in Romania (Mitkova et al., 2021; Finnish Medicines Agency Fimea, 2021; Statistics on Medicines, 2024).

There were differences in the utilization of the antiparkinsonian medication between Romania and other countries that we found. These differences may be explained by differences in population morbidity and also by the diagnosis in the early stages of the PD that may differ according to practices and guidelines applied in each country. Moreover, the decision to initiate the pharmacologic therapy may be delayed or not depending also on patient’s preferences. Population’s access to drugs, the costs of drugs and the effect of information and regulatory measures are among other causes for these differences (Björn et al., 2016).

## 5 Strengths and limitations

The present study relies on drug utilization data since the adoption of the social health insurance system in Romania (1998), showing essential insights into the utilization of antiparkinsonian medication over time. Another strong point is the data originating from CEGEDIM, the drug utilization data provider, regulated by law, for the reports and analysis conducted by National Agency for Medicines and Medical Devices of Romania (Legislatie, 2024b). Such wide interval for data availability (25 years) was not covered until present in Romania drug utilization published research. By using the ATC/DDD methodology, we were able to estimate antiparkinsonian medication usage in Romania and compare it with other countries. Analysis of individual patient-level data or prescription data would have provided valuable additional insights regarding antiparkinsonian drug use in Romania, but this data was not available.

However, it must be considered that there were national estimated drug utilization data, in aggregated form, with limited access to other variables related to drug utilization. Data from CEDEDIM panel might have included products with uneven distribution (e.g., products dispatched only in some pharmacies) with higher error margins. Also, for the new medicines, atypical sales could be observed due to supply chain phenomena. The main limitation of our study is the lack of individual-level patient data and no information on indications. Some of the antiparkinsonian medications were being used for reasons other than treating Parkinson’s disease, such as trihexyphenidyl or amantadine or even off-label such as selegiline for depression.

## 6 Conclusion

The overall trend for antiparkinsonian drug consumption in Romania showed a steady increase during 1998–2022, likely reflecting long-term patterns in usage despite short-term fluctuations in the data that were not statistically significant.

Utilization of levodopa and its combinations increased continuously, becoming the most widely used antiparkinsonian medication in Romania from 2005 until the end of the study. We noticed that the four significant reductions in antiparkinsonian medications aligned with the pandemic but also with three major changes in the Romanian healthcare system. This suggests that administrative changes may have a detrimental impact on medication prescribing and subsequently on PD patients’ therapy. The identification of prescribing trends regarding antiparkinsonian medication use in Romania can provide actionable insights for improving healthcare delivery. Future studies are necessary to understand the factors (including medication safety) that influence antiparkinsonian medication utilization in Romania.

## Data Availability

The data analyzed in this study is subject to the following licenses/restrictions: Data was provided by CEGEDIM Romania. Requests to access these datasets should be directed to cfarah@umfcluj.ro.
