# Supplementary material for: Drug utilization study of antiparkinsonian medication in Romania during 25 years
Source: Front Pharmacol. 2025 Feb 5;16:1534344. doi: 10.3389/fphar.2025.1534344 (PMC11835688; doi:10.3389/fphar.2025.1534344)
Supplement: Supplementary file 1 [file Supplementaryfile1.pdf]

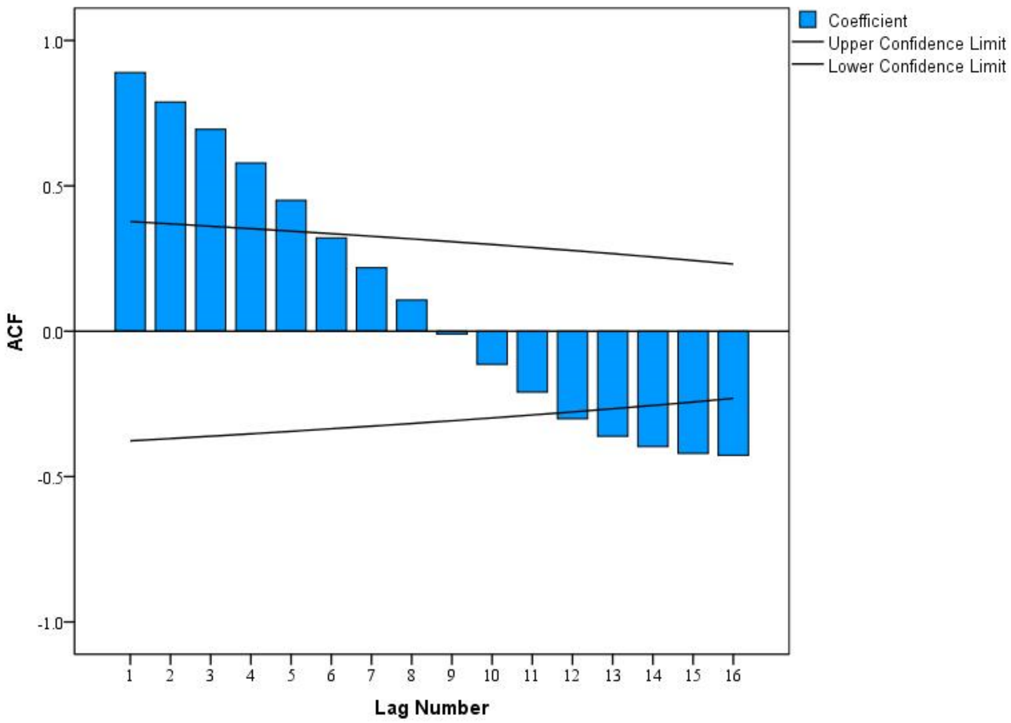

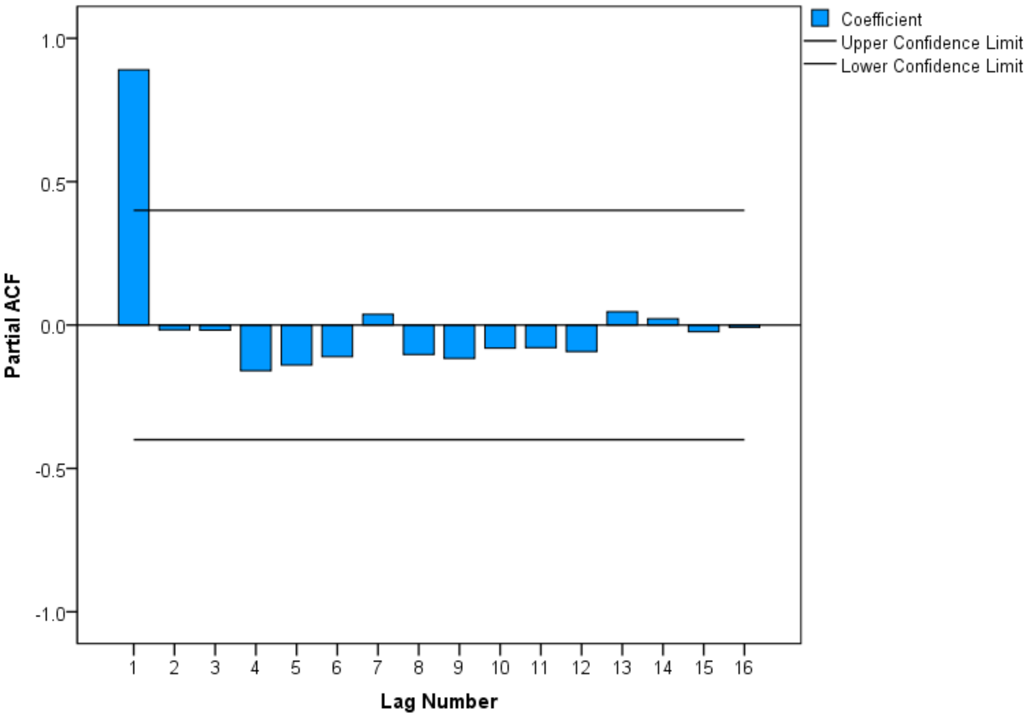

Figure S1. Autocorrelation parameters ACF and PACF of the original time series, before performing any differentiation.

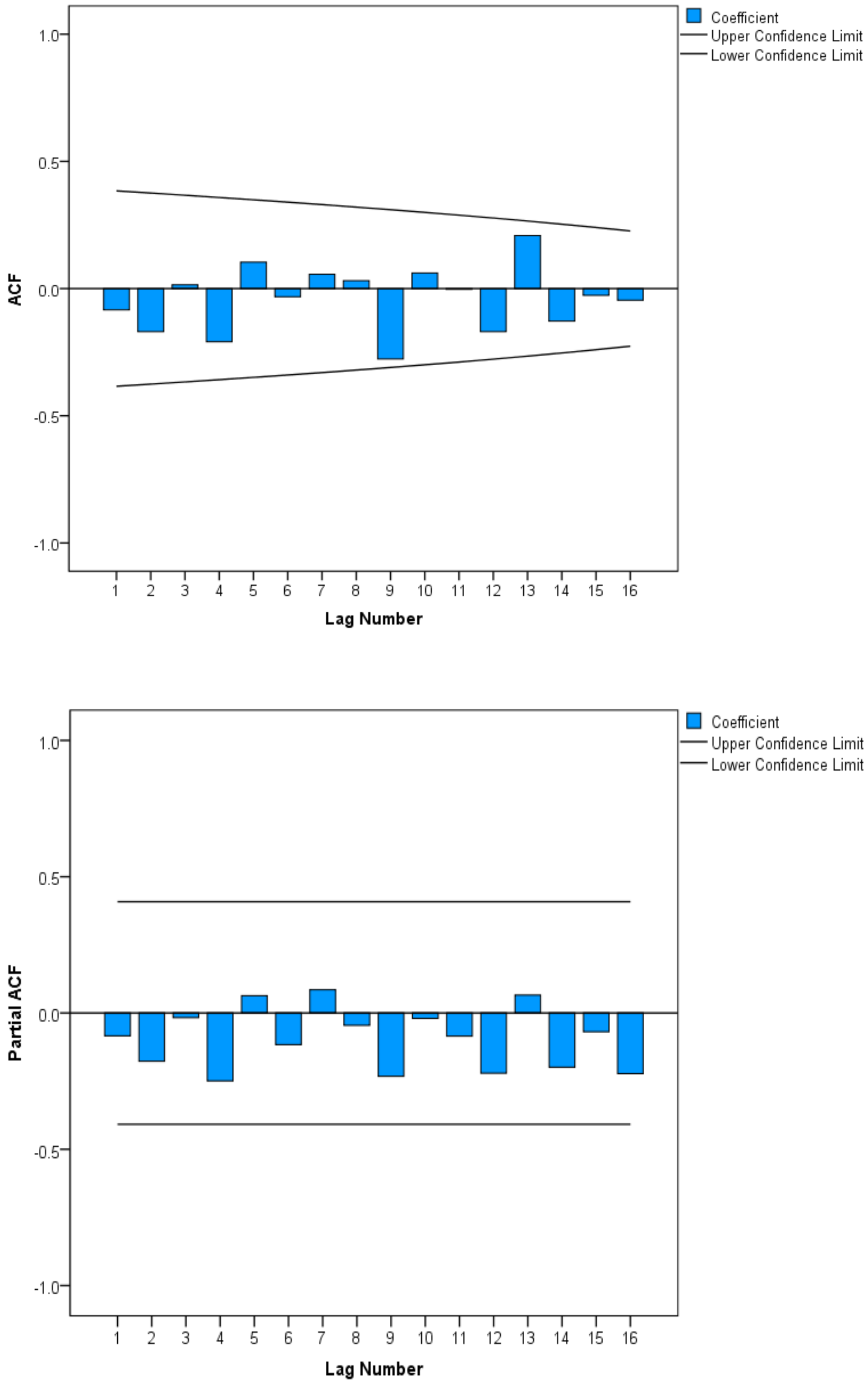

Figure S2. Autocorrelation parameters ACF and PACF after performing one differentiation. Significant lags, characterised by exceeding the black line, are considered as possible values for ARIMA-model.

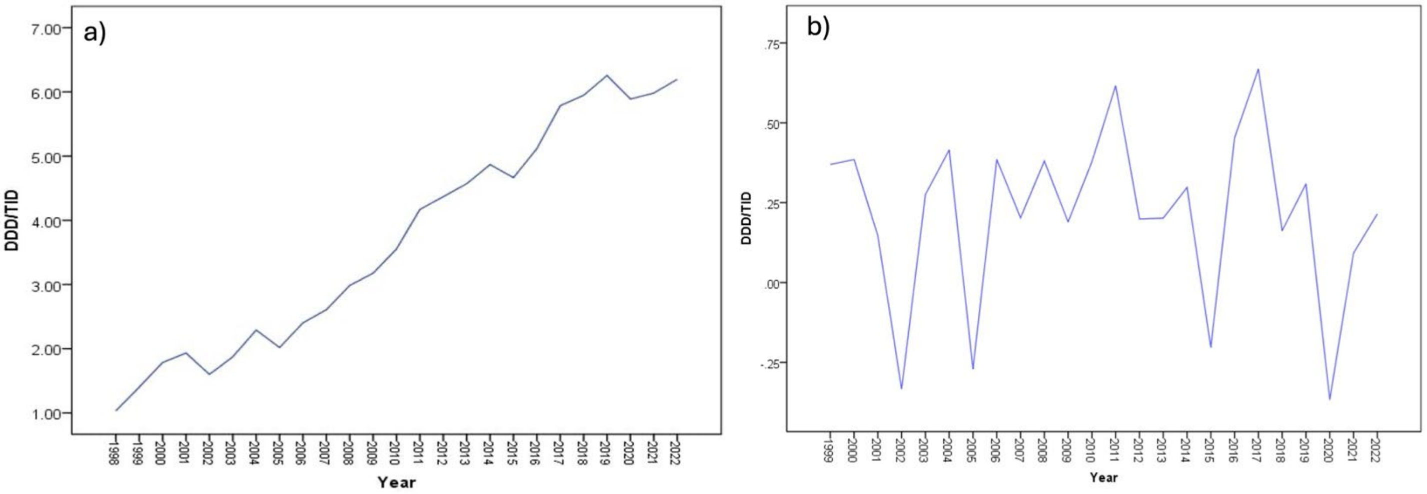

Figure S3. Time-series of antiparkinsonian drugs utilization (expressed as DDD/TID) between 1998 – 2022 a) and the series after single differentiating b).
